# Ressonância Magnética Cardíaca em Campo Magnético de 7 Tesla: Experiência Inicial com os Núcleos de Hidrogênio e do Sódio

**DOI:** 10.36660/abc.20220762

**Published:** 2023-07-27

**Authors:** Carlos E. Rochitte, Douglas C. Silva, Maria C. Otaduy, Khallil T. Chaim, Cesar H. Nomura, Bruno Caramelli

**Affiliations:** 1 Hospital das Clínicas Faculdade de Medicina Universidade de São Paulo São Paulo SP Brasil Instituto do Coração (Incor) do Hospital das Clínicas da Faculdade de Medicina da Universidade de São Paulo (HCFMUSP) – Setor de Ressonância Magnética e Tomografia Computadorizada Cardiovascular do, São Paulo, SP – Brasil; 2 Hospital das Clínicas Faculdade de Medicina Universidade de São Paulo São Paulo SP Brasil Hospital das Clínicas da Faculdade de Medicina da Universidade de São Paulo (HCFMUSP) – Departamento de Radiologia e Oncologia do, São Paulo, SP – Brasil; 3 Hospital das Clínicas Faculdade de Medicina Universidade de São Paulo São Paulo SP Brasil Instituto do Coração (Incor) do Hospital das Clínicas da Faculdade de Medicina da Universidade de São Paulo (HCFMUSP) – Unidade de Medicina Interdisciplinar em Cardiologia, São Paulo, SP – Brasil

**Keywords:** Ressonância Magnética Cardíaca (RMC, Campo Ultra-alto (UHF, 7Tesla, Imagem de sódio (23Na

## Introdução

A integridade celular do tecido do miocárdio é uma informação crucial para avaliar a viabilidade do coração.^1^ A ressonância magnética cardíaca (RMC) é considerada o método padrão-ouro na análise funcional do coração e desempenha um papel essencial no diagnóstico de várias cardiomiopatias, incluindo infarto do miocárdio.^2^ Quando há uma falha no suprimento energético das células cardíacas, ocorre uma diminuição do fluxo sanguíneo em determinadas regiões, o que pode levar à morte celular. A ressonância magnética é capaz de avaliar a viabilidade do tecido cardíaco por meio da interação com os prótons presentes nesse tecido, sendo o núcleo de hidrogênio o mais comum para esse propósito.^3^

Nos últimos anos outro núcleo surgiu como possível marcador isquêmico do coração, o sódio. Trabalhos realizados em animais mostraram que a evolução do processo isquêmico tem correlação direta com o acúmulo de sódio intracelular.^4^ Neste processo isquêmico a função da bomba de sódio-potássio é comprometida gerando um desequilíbrio na concentração deste eletrólito,^1 , 5 , 6^ levando ao influxo de sódio para o meio intracelular. Os estudos sugerem também que a concentração de sódio está diretamente relacionada à extensão de lesão isquêmica.^7^ Resumidamente, a concentração do sódio é maior no meio extracelular na situação fisiológica normal, porém com a falha na função da bomba de sódio-potássio há um influxo de sódio para o meio intracelular tornando-se então, um potencial marcador para eventos isquêmicos no coração.^1 , 5 , 6 , 8^

A RMC usa núcleos de hidrogênio devido à alta disponibilidade e características físicas do próton único que constitui este núcleo. O sódio (^23^Na) começou a ser explorado como núcleo biomarcador em animais, mas apresentava algumas limitações, como a baixa concentração e características giromagnéticas, principalmente em equipamentos disponíveis para uso clínico de 1,5 e 3 Tesla (T).^1 , 4 , 7^ Com o advento de ressonâncias de ultra-alto campo, 4,7 e 7 T, essas limitações puderam ser mitigadas, expandindo ainda mais o potencial na avaliação do coração por ressonância magnética.^2 , 4 , 7 , 8^

**Figura 1:**
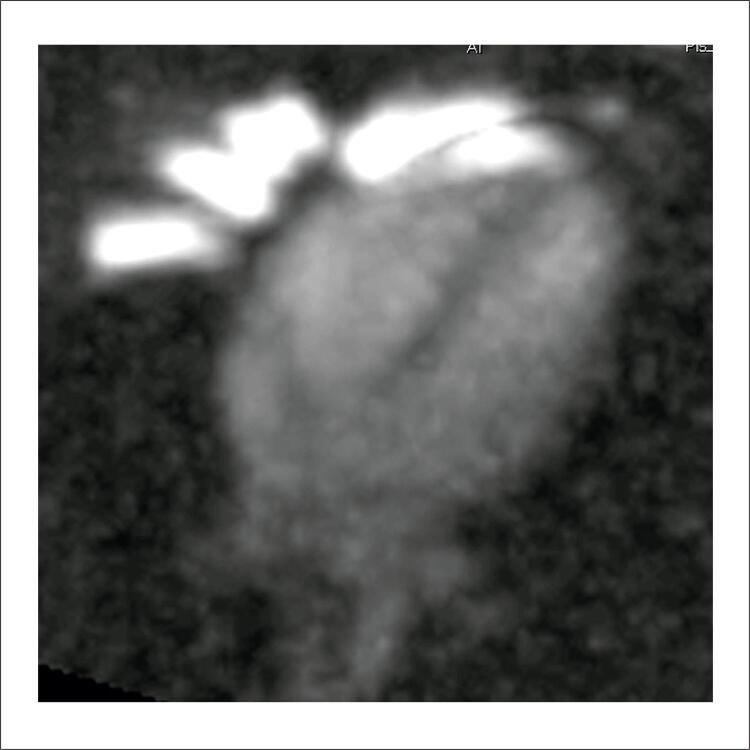
– Imagem de eixo longo do coração de voluntário humano, no plano 4 câmaras, adquirida em ressonância magnética de ultra-alto campo (7 Tesla), mostrando concentração de sódio no coração. Observa-se a concentração maior de sódio no sangue (branco) que no miocárdio (escuro).

**Figura 2:**
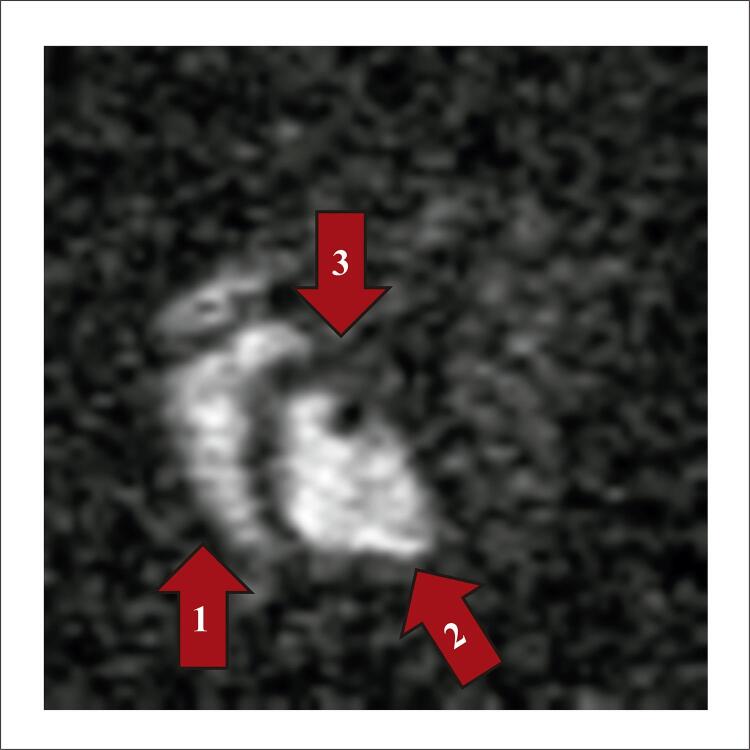
– Imagem de sódio eixo curto do coração de cão em ressonância de 4,7 Tesla. Setas: 1. Ventrículo direito. 2. Ventrículo esquerdo. 3. Septo interventricular. (Arquivo Pessoal Rochitte et al.4).

**Figura 3:**
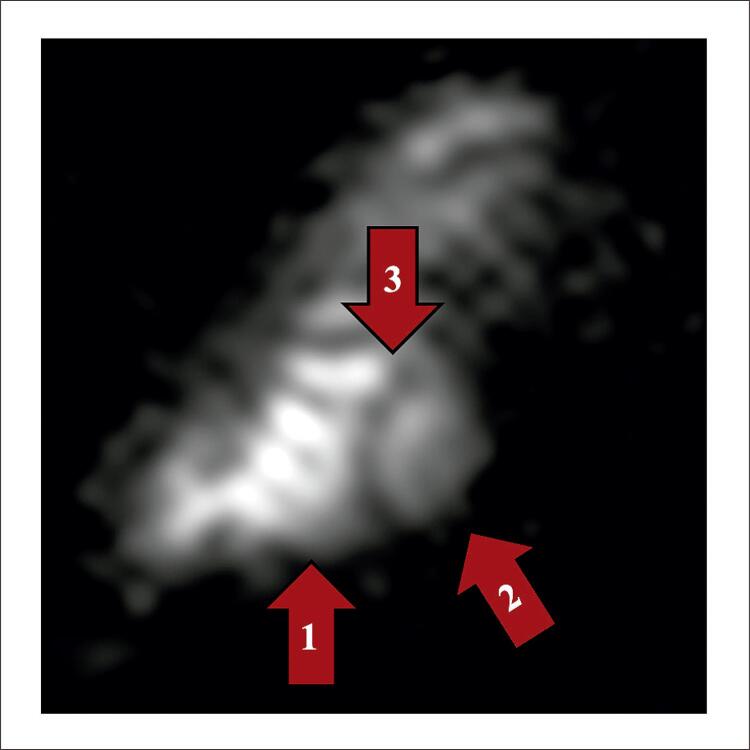
– Imagem de sódio eixo curto do coração de voluntário humano em ressonância de 3 Tesla. Setas: 1. Ventrículo direito. 2. Ventrículo esquerdo. 3. Septo interventricular. (Arquivo pessoal Gai et al.13).

Pesquisas comprovam a capacidade de avaliar a concentração de sódio ( Figura 1 ) e de obter imagens do sódio com maior resolução espacial, causada pelo maior campo magnético que gera maior sinal, permitindo então a reconstrução de imagens com maior definição anatómica.^2 , 8 - 10^ Outros estudos demonstraram as bases teóricas da possibilidade do uso clínico da imagem de sódio na avaliação do miocárdio em equipamento de 1,5 T.^11 , 12^ Rochitte et al. em 2000 demonstraram a correlação entre o acúmulo de sódio e necrose miocárdica em animais utilizando equipamento de 4,7 T.^4^ O estudo demonstrou também a correlação entre a viabilidade do tecido e a concentração de sódio, onde miócitos não viáveis demonstraram um acúmulo de sódio ( Figura 2 ). Em trabalho subsequente realizado no National Institute of Health, foram realizadas imagens de sódio de voluntários em equipamento de ressonância magnética de uso clínico de 3,0 T de campo magnético.^1^ Exemplos das imagens obtidas estão na Figura 3 .^13^

**Figura 4:**
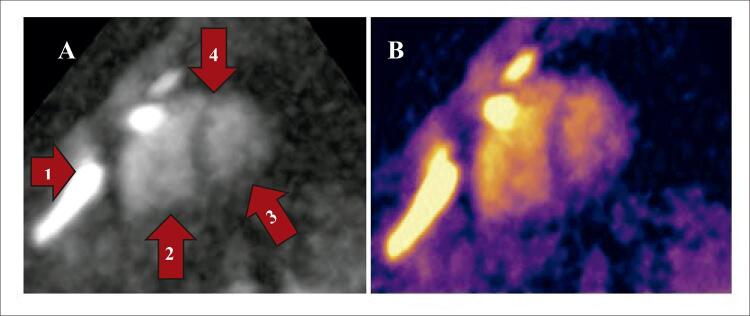
– Painel A) Imagem de sódio eixo curto do coração de voluntário humano em ressonância de 7 Tesla. Setas: 1. Cartilagem esternal, devido à grande quantidade de sódio vemos um hipersinal. 2. Ventrículo direito. 3. Ventrículo esquerdo. 4. Septo interventricular. Painel B) A mesma imagem com look-up table (LUT) colorida.

Atualmente, a utilização da RMC para análise da extensão de lesões isquêmicas do coração, utilizando o hidrogênio como núcleo gerador do sinal, demanda a utilização de contraste baseado em gadolínio a fim de realçar estruturas do coração e expor o espaço extracelular. O realce tardio do miocárdio com lesão definitiva acontece após 10 a 15 minutos.^13^ A vantagem das imagens de sódio se dá pela possibilidade de excluir a necessidade de utilização de contraste.^4^ Atualmente, trabalhamos com sequências de pulso específicas para análise de sódio ( Figura 4 ) modificadas para melhor visualização do sódio nos eixos cardíacos.

### Métodos – pesquisa pioneira

Em toda a América Latina existe apenas um equipamento de alto campo magnético, o Magnetom 7T (Siemens Healthineers – GhMb), introduzido fundamentalmente para pesquisa e estudos de cadáveres na Plataforma de Imagem de Sala de Autopsia (PISA).^9^ Ao longo dos últimos anos, diversos projetos de pesquisas em várias áreas do conhecimento foram iniciados, inclusive em Cardiologia.

Atualmente, estudos focados em RMC fazem parte da rotina nas pesquisas do equipamento. Os protocolos ( Tabelas 1 e 2 ) que utilizam imagens de hidrogênio e sódio vêm sofrendo modificações com o objetivo de atingir a melhor qualidade das imagens com ambos os núcleos, uma vez que os exames de coração em campos de alta intensidade, como 7 T, traz inúmeros desafios. Entre os desafios de exames cardíacos em equipamento de alto campo são a sincronização com o ciclo cardíaco, artefatos de fluxo de sangue e homogeneidade do campo magnético.^10^ Após o período de ajustes de protocolos, notou-se a evolução na qualidade das imagens ( Figura 2 ), possibilitando o potencial uso clínico para os diagnósticos de cardiomiopatias. As imagens foram coletadas com bobinas específicas de 4 canais que podem sintonizar os 2 núcleos, sódio e hidrogênio, sem a necessidade de modificar a posição do paciente ou de qualquer troca de equipamento (bobina de dupla sintonia – dual tune ^1^H/^23^Na).

**Tabela 1:** – Protocolo RMC 7T

Sequência	TR (ms)	TE (ms)	Sequência de Pulso	Trigger	Slice	Flip Angle	SNR	Tempo de aquisição
Hidrogênio	44,4	2,24	FLASH	Pulso retro	8 mm	40*	1	0,35 min
Sódio	37	1,36	GRE	Pulso retro	8 mm	112*	1	8,14 min

**Tabela 2:** – Protocolo **23** Na detalhado RMC 7 Tesla

Sistema		Rotina		Contraste	
N2	Ligado	Slab	1	Preparação magn.	Nenhuma
H3	Desligado	Espessura de slice	8,00 mm	Flip angle	112 graus
N3	Ligado	TR	37,00 ms	Supressão de gordura	Nenhum
H4	Desligado	TE	1,36 ms	Restaurar magn.	Desligado
N4	Ligado	Médias	6	Modo de média	Curto prazo
Modo de posicionamento	FIX	Concatenações	1	Reconstrução	Magnitude
MSMA	S - C - T	Filtro	Nenhuma	Medidas	1
Sagital	R >> L	Elementos da bobina	N1-4	Séries múltiplas	Desligado
Coronal	A >> P	Slabs	1	Resolução	
Transversal	F >> H	Slabs	1	Resolução base	80
Save uncombined	Desligado	Fator de dist.	0	Resolução fase	100%
Coil Combine Mode	Soma dos quadrados	Posição	L16.9 A26.4 F23.7	Resolução slice	100%
AutoAlign	---	Orientação	T > S33,0 > C-26,6	Phase partial Fourier	06/ago
Auto Coil Select	Default	Fase enc. dir.	A >> P	Slice partial Fourier	06/ago
Modo shim	Tune up	Rotação	16,18 graus	Trajetória	Cartesiana
Tolerância de ajuste	Volume de ajuste automático	Auto	Desligado	Interpolação	Desligado
! Posição	L20.0 A21.0 F12.5	Fase oversampling	0	Modo PAT	Nenhum
! Orientação	Transversal	Slice oversampling	0,00 %	Filtro de imagem	Desligado
! Rotação	0,00 graus	Slices per slab	20		
! R >> L	120 mm	FoV leitura	256 mm		
! A >> P	95 mm	FoV fase	100,0 %		
! F >> H	115 mm				

Para a captação do sinal de ambos os núcleos também é necessária uma antena de radiofrequência, ou bobina de RF, dedicada (MRI.TOOLS GmbH). O PISA conta com um modelo híbrido, dedicado a ambos os núcleos e configurado de maneira dedicada ao Magnetom 7T. A antena é composta por duas faces, uma posterior e plana, outra anterior ligeiramente curvada para melhor posicionamento anatômico. Ambas as faces são compostas por 2 espirais semi-retangulares, o maior sendo responsável pela sintonia do ^23^Na e os menores pela sintonia do ^1^H. Durante a aquisição das imagens podemos alternar o elemento com o qual queremos trabalhar e assim gerar imagens de ^1^H ou ^23^Na.^5^ A antena de RF chegou ao PISA através do laboratório de investigação médica, com o objetivo de gerar imagens morfofuncionais. Atualmente, é uma configuração única em toda a América Latina.

**Figura 5:**
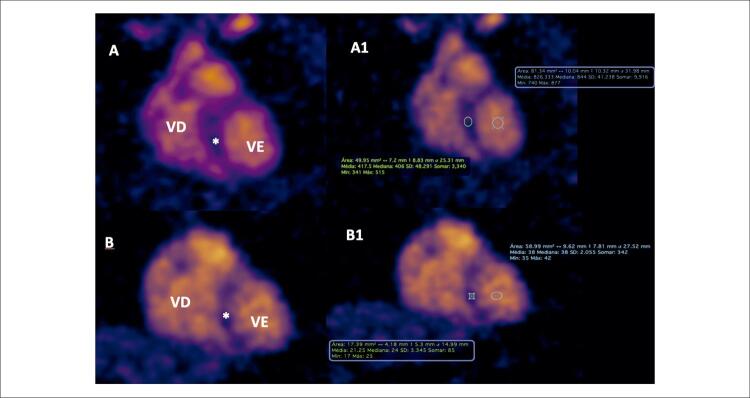
– A e B) Imagem de sódio eixo curto do coração de voluntário humano em ressonância de 7 Tesla. Na imagem A1) ROI* – RSR: 6,40 e ROIVE – RSR: 19,00. Na imagem B1) ROI* – RSR: 9.82 e ROIVE – RSR: 20,40. Imagem com look-up table (LUT) colorida. RSR = sinal / desvio padrão. O asterisco indica o septo interventricular. ROI: região de interesse; RSR: relação sinal-ruído; VD: ventrículo direito; VE: ventrículo esquerdo.

Foram realizados 4 exames em voluntários com imagens de hidrogênio e de sódio ( Tabela 3 ). Para avaliação das imagens contamos com revisão e classificação da qualidade da imagem por médico experiente, sendo: 1 – prejudicada, 2 – aceitável, 3 – adequada e 4 – excelente. O cálculo da relação sinal-ruído (RSR) foi realizado no septo interventricular ( Figura 5 ) das imagens ^23^Na, seguindo um dos métodos presente nas diretrizes da National Electrical Manufacturers Association, RSR = sinal / desvio padrão (NEMA Standards Publication MS 1-2008 R2014, R2020 - Determination of Signal-to-Noise Ratio [SNR] in Diagnostic Magnetic Resonance Imaging).

**Tabela 3:** – Voluntários para RMC com classificação da qualidade de imagem: 1 – prejudicada, 2 – aceitável, 3 – adequada, 4 – excelente. RSR = sinal / desvio padrão

Voluntários	Data do estudo	Idade (anos)	Sexo	Peso (kg)	Altura (cm)	QI	RSR VE	RSR Septo
V1	03/11/2020	31	Masc	82	184	2	19,00	6,40
V2	10/11/2020	47	Masc	94	188	3	9,72	20,40
V3	24/11/2020	29	Fem	64	170	3	15,00	36,60
V4	15/12/2020	35	Fem	61	172	4	19,79	11,94

QI: qualidade da imagem; RSR: relação sinal-ruído; VE: ventrículo esquerdo.

### Imagens de sódio (23Na)

As primeiras impressões com esse tipo de exame mostraram que os desafios descritos pela literatura em ressonância cardíaca de ultra-alto campo são, de fato, fatores limitantes, especialmente nas imagens de sódio. Devido às características giromagnéticas e disponibilidade do sódio, chegamos a um complexo trabalho para manter a homogeneidade do campo magnético. Apesar das limitações e desafios, foi possível gerar imagens de sódio e hidrogênio com qualidade suficiente para a avaliação anatômica e concentração de sódio nas cavidades e músculo cardíaco. De forma qualitativa, vimos também uma relação de muito sinal no sangue, onde temos maior concentração de ^23^Na e pouco sinal no septo interventricular ( Figura 5 ), em comparação com situações normais.^4^ Com esta pesquisa, abrimos um leque de possibilidades para futuros trabalhos. Com uma amostragem maior de pacientes, poderemos validar o método para uso clínico e de pesquisa em humanos. Dentre as possíveis aplicações futuras para esta técnica, podemos citar a identificação de lesões miocárdicas preliminares ou incipientes, secundárias a agressões por medicamentos cardiotóxicos, isquemia, inflamação ou infecção. Novas imagens e mecanismos podem ser identificados para processos inflamatórios crônicos e progressivos, como os que ocorrem em processos autoimunes ou infecções crônicas. Apesar dos desafios inerentes à realização da RMC em 7 T, nosso trabalho demonstra que é possível gerar imagens utilizando hidrogênio para avaliar a anatomia cardíaca. Além disso, validamos que as imagens geradas com sódio podem ser correlacionadas com a anatomia do coração. Esse resultado abre caminho para estudos futuros e pode potencialmente facilitar a utilização clínica da RMC de ultra-alto campo.
