# Supplementary material for: Ressonância Magnética Cardíaca em Campo Magnético de 7 Tesla: Experiência Inicial com os Núcleos de Hidrogênio e do Sódio
Source: Arq Bras Cardiol. 2023 Jul 27;120(7):e20220762. [Article in Portuguese] doi: 10.36660/abc.20220762 (PMC10382146; doi:10.36660/abc.20220762)
Supplement: Translation

# Translation: Cardiac Magnetic Resonance Imaging in a 7 Tesla Magnetic Field: Initial Experience with Hydrogen and Sodium Nuclei

**Authors:** Carlos E. Rochitte, Douglas C. Silva, Maria C. Otaduy, Khallil T. Chaim, Cesar H. Nomura, Bruno Caramelli

**Affiliations:** 1 Brasil Instituto do Coração (Incor) do Hospital das Clínicas da Faculdade de Medicina da Universidade de São Paulo (HCFMUSP) – Setor de Ressonância Magnética e Tomografia Computadorizada Cardiovascular do, São Paulo, SP – Brasil; 2 Brasil Hospital das Clínicas da Faculdade de Medicina da Universidade de São Paulo (HCFMUSP) – Departamento de Radiologia e Oncologia do, São Paulo, SP – Brasil; 3 Brasil Instituto do Coração (Incor) do Hospital das Clínicas da Faculdade de Medicina da Universidade de São Paulo (HCFMUSP) – Unidade de Medicina Interdisciplinar em Cardiologia, São Paulo, SP – Brasil

**Keywords:** Cardiac Magnetic Resonance (CMR, Ultra-high Field (UHF, 7 Tesla, Sodium Image (23Na

## Introduction

The cellular integrity of myocardial tissue is crucial information to evaluate the viability of the heart.^1^ Cardiac magnetic resonance imaging (CMR) is considered the gold standard method for functional analysis of the heart, and it plays an essential role in the diagnosis of several cardiomyopathies, including myocardial infarction.^2^ When there is a failure in the energy supply of heart cells, there occurs a decrease in blood flow in certain regions, which can lead to cell death. CMR can assess the viability of cardiac tissue by means of interaction with the protons present in this tissue, the hydrogen nucleus being the most common for this purpose.^3^

In recent years, another nucleus has emerged as a possible ischemic marker of the heart, namely, sodium. Studies carried out in animals have shown that the evolution of the ischemic process is directly correlated with the accumulation of intracellular sodium.^4^ In this ischemic process, the function of the sodium-potassium pump is compromised, generating an imbalance in the concentration of this electrolyte,^1 , 5 , 6^ leading to influx of sodium into the intracellular environment. Studies also suggest that sodium concentration is directly related to the extent of ischemic injury.^7^ Sodium concentration is higher in the extracellular environment in normal physiological situations, but with failure in the function of the sodium-potassium pump, there is an influx of sodium into the intracellular environment, thus making it a potential marker for ischemic events in the heart.^1 , 5 , 6 , 8^

CMR uses hydrogen nuclei due to the high availability and physical characteristics of the single proton that constitutes this nucleus. Sodium (^23^Na) began to be explored as a core biomarker in animals, but it showed some limitations, such as low concentration and gyromagnetic characteristics, mainly in equipment available for clinical use at 1.5 and 3 Tesla (T).^1 , 4 , 7^ With the advent of ultra-high-field resonance at 4.7 and 7 T, these limitations could be mitigated, further expanding the potential of CMR.^2 , 4 , 7 , 8^

Research has demonstrated that it is possible to assess sodium concentration ( Figure 1 ) and obtain sodium imaging with greater spatial resolution, due to the greater magnetic field that generates a higher signal, thus allowing the reconstruction of images with greater anatomical definition.^2 , 8 - 10^ Other studies have demonstrated the theoretical bases for the possibility of clinical use of sodium imaging in the assessment of the myocardium using 1.5 T equipment.^11 , 12^ In 2000, Rochitte et al. demonstrated a correlation between sodium accumulation and myocardial necrosis in animals using 4.7 T equipment.^4^ The study also demonstrated the correlation between tissue viability and sodium concentration, where non-viable myocytes demonstrated an accumulation of sodium ( Figure 2 ). In subsequent research carried out at the National Institute of Health, sodium imaging of volunteers was performed in magnetic resonance equipment for clinical use with a 3.0 T magnetic field.^1^Figure 3 displays examples of the images obtained.^13^

**Figure 1:** – Long-axis imaging of the heart of a human volunteer, in the 4-chamber view, acquired in ultra-high-field magnetic resonance imaging (7 Tesla), showing sodium concentration in the heart. A higher concentration of sodium was observed in the blood (white) than in the myocardium (dark).

**Figure 2:** – Short-axis sodium imaging of the heart of a dog at 4.7 Tesla. Arrows: 1. Right ventricle. 2. Left ventricle. 3. Interventricular septum. (Personal archive of Rochitte et al. ^4^ ).

**Figure 3:** – Short-axis sodium imaging of the heart of a human volunteer at 3 Tesla. Arrows: 1. Right ventricle. 2. Left ventricle. 3. Interventricular septum. (Personal archive Gai et al. ^13^ ).

Currently, the use of CMR to analyze the extent of ischemic heart lesions, with hydrogen as the signal-generating nucleus, requires the use of gadolinium-based contrast to enhance heart structures and reveal the extracellular space. Delayed enhancement of the myocardium with definitive damage occurs after 10 to 15 minutes.^13^ The advantage of sodium imaging is that it makes it possible exclude the need for contrast.^4^ Currently, we work with specific pulse sequences for sodium analysis ( Figure 4 ), modified for better visualization of sodium in the cardiac axes.

**Figure 4:** – Panel A) Short-axis sodium imaging of the heart of a human volunteer at 7 Tesla. Arrows: 1. Sternal cartilage; due to the large amount of sodium, a hypersignal is observed. 2. Right ventricle. 3. Left ventricle. 4. Interventricular septum. Panel B) The same image with color look-up table.

### Methods – pioneering research

In all of Latin America, the Magnetom 7T (Siemens Healthineers – GhMb) is the only high magnetic field equipment; it was essentially introduced for cadaver studies and research in the Autopsy Room Imaging Platform (PISA, acronym in Portuguese).^9^ Over the past few years, diverse research projects in various areas of knowledge have been initiated, including cardiology.

Currently, studies focusing on CMR are part of the routine in research with this equipment. The protocols ( Tables 1 and 2 ) that use hydrogen and sodium imaging have undergone modifications with the objective of achieving the best image quality with both nuclei, given that cardiac exams in high-intensity fields, such as 7 T, pose numerous challenges. The challenges of cardiac examinations in high-field equipment include synchronization with the cardiac cycle, blood flow artifacts, and homogeneity of the magnetic field.^10^ After the period of protocol adjustments, an evolution in the image quality was observed ( Figure 2 ), allowing the potential clinical use for diagnosing cardiomyopathies. Images were acquired with specific 4-channel coils that can tune both nuclei, sodium and hydrogen, without needing to modify the patient’s position or change equipment (dual-tune^1^H/^23^Na coil).

**Table 1:** – Protocol for cardiac magnetic resonance imaging at 7 Tesla

Sequence	TR (ms)	TE (ms)	Pulse sequence	Trigger	Slice	Flip Angle	SNR	Acquisition time
Hydrogen	44.4	2.24	FLASH	Retro pulse	8 mm	40*	1	0.35 min
Sodium	37	1.36	GRE	Retro pulse	8 mm	112*	1	8.14 min

**Table 2:** – Detailed protocol for **23** Na cardiac magnetic resonance imaging at 7 Tesla

System		Routine		Contraste	
N2	On	Slab	1	Magn. Preparation	None
H3	Off	Slice thickness	8.00 mm	Flip angle	112 deg
N3	On	TR	37.00 ms	Fat suppr.	None
H4	Off	TE	1.36 ms	Restore magn.	Off
N4	On	Averages	6	Averaging mode	Short term
Positioning mode	FIX	Concatenations	1	Reconstruction	Magnitude
MSMA	S - C - T	Filter	None	Measurements	1
Sagittal	R >> L	Coil elements	N1-4	Multiple series	Off
Coronal	A >> P	Slabs	1	Resolution	
Transversal	F >> H	Slabs	1	Base resolution	80
Save uncombined	Off	Dist. factor	0	Phase resolution	100%
Coil Combine Mode	Sum of Squares	Position	L16.9 A26.4 F23.7	Slice resolution	100%
AutoAlign	---	Orientation	T > S33.0 > C-26.6	Phase partial Fourier	06/ago
Auto Coil Select	Default	Phase enc. dir.	A >> P	Slice partial Fourier	06/ago
Shim mode	Tune up	Rotation	16.18 deg	Trajectory	Cartesian
Adjustment Tolerance	Auto Adjust volume	Auto	Off	Interpolation	Off
! Position	L20.0 A21.0 F12.5	Phase oversampling	0	PAT mode	None
! Orientation	Transversal	Slice oversampling	0.0 %	Image Filter	Off
! Rotation	0.00 deg	Slices per slab	20		
! R >> L	120 mm	FoV read	256 mm		
! A >> P	95 mm	FoV phase	100.0 %		
! F >> H	115 mm				

To obtain the signal of both nuclei, a dedicated radiofrequency antenna or coil (MRI.TOOLS GmbH) is also necessary. PISA has a hybrid model, dedicated to both nuclei and configured specifically for Magnetom 7T. The antenna is composed of 2 faces, one posterior and flat and another anterior slightly curved for better anatomical positioning. Both faces are composed of 2 semi-rectangular spirals, the larger being responsible for^23^Na tuning and the smaller for^1^H tuning. During image acquisition, we can alternate the element with which we wish to work and thus generate^1^H or^23^Na images.^5^ The radiofrequency antenna first arrived at PISA through the medical research laboratory, with the objective of generating morphofunctional images. Currently, it is the only configuration of its type in all of Latin America.

We performed 4 exams on volunteers with hydrogen and sodium imaging ( Table 3 ). To evaluate the images, we relied on review and classification of image quality by an experienced doctor, as follows: 1 – impaired, 2 – acceptable, 3 – adequate, and 4 – excellent. The calculation of the signal-to-noise ratio (SNR) was performed in the interventricular septum ( Figure 5 ) of the^23^Na images, following one of the methods present in the guidelines of the National Electrical Manufacturers Association, where SNR is equal to signal divided by standard deviation (NEMA Standards Publication MS 1-2008 R2014, R2020 - Determination of Signal-to-Noise Ratio [SNR] in Diagnostic Magnetic Resonance Imaging).

**Table 3:** – Volunteers for cardiac magnetic resonance imaging with classification of image quality: 1 – impaired, 2 – acceptable,3 – adequate, 4 – excellent. SNR calculated using the following equation: SNR = signal / standard deviation

Volunteers	Study date (day/month/year)	Age (years)	Sex	Weight (kg)	Height (cm)	IQ	SNR LV	SNR Septum
V1	03/11/2020	31	Male	82	184	2	19.00	6.40
V2	10/11/2020	47	Male	94	188	3	9.72	20.40
V3	24/11/2020	29	Female	64	170	3	15.00	36.60
V4	15/12/2020	35	Female	61	172	4	19.79	11.94

IQ: image quality; LV: left ventricle; SNR: signal-to-noise ratio.

**Figure 5:** – A and B) Short-axis sodium imaging of the heart of a human volunteer at 7 Tesla. In image A1) ROI* – SNR: 6.40 and ROI LV – SNR: 19.00. In image B1) ROI* – SNR: 9.82 and ROI LV – SNR: 20.40. Image with color look-up table. SNR calculated using the following equation: SNR = signal / standard deviation. The asterisk indicates the interventricular septum. LV: left ventricle; ROI: region of interest; RV: right ventricle; SNR: signal-to-noise ratio.

### Sodium imaging (23Na)

The initial impressions with this type of exam have shown that the challenges described in the literature regarding ultra-high-field CMR are, in fact, limiting factors, especially in sodium imaging. Due to the gyromagnetic characteristics and sodium availability, we face the complex task of maintaining the homogeneity of the magnetic field. Despite the limitations and challenges, it was possible to generate sodium and hydrogen images with sufficient quality for anatomical assessment and sodium concentration in the cardiac chambers and muscle. Qualitatively, we also observed a relationship with a high signal in the blood, where there is a higher concentration of^23^Na, and little signal in the interventricular septum ( Figure 5 ), compared to normal situations.^4^ This research has opened a range of possibilities for future studies. With a larger sample of patients, we will be able to validate the method for clinical use and research in humans. The possible future applications for this technique include the identification of preliminary or incipient myocardial lesions, secondary to injury due to cardiotoxic medications, ischemia, inflammation, or infection. New images and mechanisms may be identified for chronic and progressive inflammatory processes, such as those that occur in autoimmune processes or chronic infections. Despite the challenges inherent in performing CMR at 7 T, our study has demonstrated that it is possible to generate images using hydrogen to assess cardiac anatomy. Furthermore, we have confirmed that images generated with sodium can be correlated with the anatomy of the heart. This result paves the way for future studies and potentially facilitates the clinical use of ultra-high-field CMR.
